# Control of Cerebellar Long-Term Potentiation by P-Rex-Family Guanine-Nucleotide Exchange Factors and Phosphoinositide 3-Kinase

**DOI:** 10.1371/journal.pone.0011962

**Published:** 2010-08-04

**Authors:** Claire Jackson, Heidi C. Welch, Tomas C. Bellamy

**Affiliations:** 1 Laboratory of Molecular Signalling, The Babraham Institute, Cambridge, United Kingdom; 2 Inositide Laboratory, The Babraham Institute, Cambridge, United Kingdom; 3 School of Biomedical Sciences, University of Nottingham, Nottingham, United Kingdom; Vrije Universiteit Amsterdam, Netherlands

## Abstract

**Background:**

Long-term potentiation (LTP) at the parallel fibre–Purkinje cell synapse in the cerebellum is a recently described and poorly characterized form of synaptic plasticity. The induction mechanism for LTP at this synapse is considered reciprocal to “classical” LTP at hippocampal CA1 pyramidal neurons: kinases promote increased trafficking of AMPA receptors into the postsynaptic density in the hippocampus, whereas phosphatases decrease internalization of AMPA receptors in the cerebellum. In the hippocampus, LTP occurs in overlapping phases, with the transition from early to late phases requiring the consolidation of initial induction processes by structural re-arrangements at the synapse. Many signalling pathways have been implicated in this process, including PI3 kinases and Rho GTPases.

**Principal Findings:**

We hypothesized that analogous phases are present in cerebellar LTP, and took as the starting point for investigation our recent discovery that P-Rex – a Rac guanine nucleotide exchange factor which is activated by PtdIns(3,4,5)P_3_ – is highly expressed in mouse cerebellar Purkinje neurons and plays a role in motor coordination. We found that LTP evoked at parallel fibre synapses by 1 Hz stimulation or by NO donors was not sustained beyond 30 min when P-Rex was eliminated or Rac inhibited, suggesting that cerebellar LTP exhibits a late phase analogous to hippocampal LTP. In contrast, inhibition of PI3 kinase activity eliminated LTP at the induction stage.

**Conclusions:**

Our data suggest that a PI3K/P-Rex/Rac pathway is required for late phase LTP in the mouse cerebellum, and that other PI3K targets, which remain to be discovered, control LTP induction.

## Introduction

The cerebellar cortex controls fine motor coordination and associative learning. Most computational models of cerebellar function are based on learning-induced changes in the strength of transmission at parallel fibre-Purkinje neuron synapses [1]–[3], a process generally assumed to be achieved by frequency-dependent long-term synaptic plasticity [4]–[7].

Long-term plasticity occurs as two major forms, which reverse one another, long term depression (LTD) and long-term potentiation (LTP). LTD at the parallel fibre synapse has been extensively studied, and its molecular basis is the increased phosphorylation of GluR2 subunits of AMPA receptors in the postsynaptic density, promoting receptor internalization (for detailed review see [8]). LTD can be evoked by co-stimulation of parallel fibre and climbing fibre inputs (typically at 1 Hz), causing high amplitude Ca^2+^ increases, and activation of metabotropic glutamate and nitric oxide receptors. Through activation of CaM kinase II, protein kinase C, and cGMP-dependent protein kinase, these signalling pathways cooperatively increase phosphorylation of GluR2 subunits, accelerating receptor internalization and thereby decreasing the strength of response to presynaptic transmitter release [8].

In contrast, the converse mechanism to postsynaptic LTD – postsynaptic LTP – has only recently been defined. LTP can be evoked by stimulation of parallel fibres alone at 1 Hz [5], [9]. A relatively modest Ca^2+^ influx generated in the absence of the climbing fibre input activates protein phosphatases (principally calcineurin; [10]), reversing GluR2 phosphorylation and reducing the rate of AMPAR internalization. Additionally, NO synthesis is required for LTP induction, in this case acting in a guanylyl cyclase-independent manner, putatively through nitrosation of NSF to promote insertion of receptors into the plasma membrane [11]. Thus, the balance between kinase and phosphatase activity determines the rate of AMPA receptor trafficking into and out of the postsynaptic density, and thereby the strength of transmission at the synapse.

This outline mechanism for induction of cerebellar LTP differs substantially from that of classical LTP in the hippocampus [12]. Hippocampal LTP is triggered by high amplitude Ca^2+^ influx through NMDA receptors, activating CaM kinase II, which phosphorylates GluR1 subunits, increasing the conductance of the AMPAR, and accelerating their insertion into the postsynaptic density [13], [14]. Thus, induction of long-term plasticity at hippocampal and cerebellar synapses has been described as having reciprocal dependence on Ca^2+^ concentration and AMPA receptor phosphorylation [12].

After induction, hippocampal LTP can be ablated by low-frequency stimulation or adenosine application [15], revealing that the initial induction phase must be reinforced by later phases of signalling in order for LTP to be consolidated. Many signalling pathways have been implicated in the expression and maintenance of hippocampal LTP, and the transition from early to late phases [16], [17], but a unifying hypothesis has been proposed that these signalling pathways ultimately converge on the modulation of actin cytoskeletal re-arrangements, which underlie morphological changes in spine structure that mediate consolidation of LTP [18]. These structural changes can occur at the level of the postsynaptic density, or even the gross morphology and number of dendritic spines. Several Rho GTPases have been implicated in the modification of cytoskeletal structure during late phase LTP, notably RhoA, Rac and Cdc42 [19], [20]. Whether such late phase processes occur in cerebellar LTP is unknown.

We sought to examine this issue, and our starting point was the recent discovery that P-Rex family Rac GEFs are expressed at high levels in cerebellar Purkinje neurons [21]. P-Rex is activated by PtdIns(3,4,5)P_3_, the product of the reaction catalysed by Class I PI3 kinases (PI3K), which have been reported to be essential for expression of hippocampal LTP [22]–[24] and for the NMDA-evoked exocytosis of GluR1-containing AMPARs in cultured hippocampal neurons [25]. P-Rex loss is also associated with changes in Purkinje neuron dendrite morphology [21], implicating it in structural changes in these postsynaptic compartments. We therefore hypothesized that P-Rex could act as an intermediary for the PI3K-dependent activation of Rac – two enzymes which have been implicated in late phase LTP in the hippocampus – and may thereby play a role in cerebellar LTP.

We found that postsynaptic LTP could be induced, but not sustained, in P-Rex-deficient Purkinje neurons. Similar results were found when Rac was inhibited, but inhibition of PI3K activity additionally compromised the early induction phase, resulting in failure to exhibit LTP at any stage. We propose that LTP at parallel fibre to Purkinje cell synapses exhibits multiple phases, and identify a PI3K/P-Rex/Rac signalling axis as a late phase mechanism for consolidation of LTP.

## Results

### Synaptic transmission, short-term plasticity, and passive membrane properties are normal in P-Rex-deficient Purkinje neurons

P-Rex elimination results in morphological changes in Purkinje neuron dendrite structure. In the first instance, therefore, we explored the impact of P-Rex deficiency on excitability and normal synaptic transmission in Purkinje neurons.

**Figure 1:**
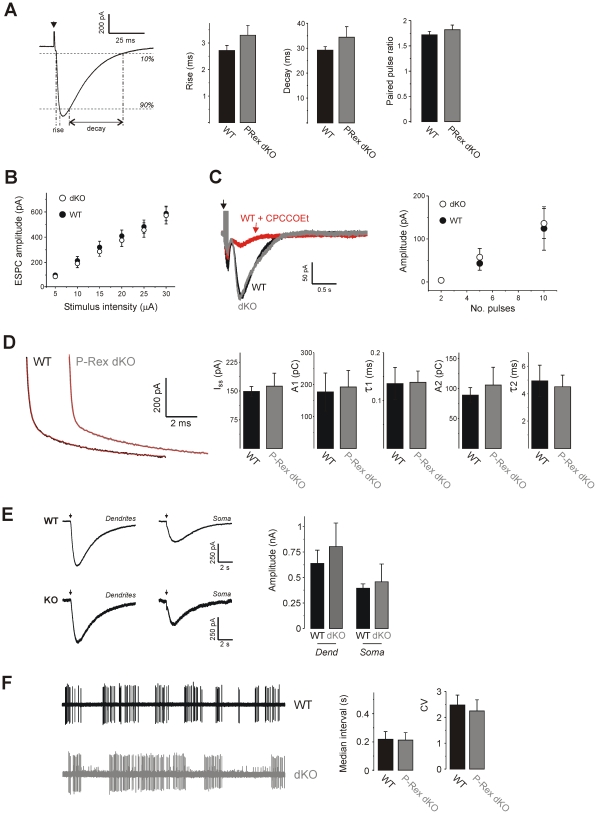
P-Rex-deficient Purkinje neurons have normal passive membrane properties and basal synaptic transmission. **A**) Rise time and decay time of EPSC (measured between 10–90% of amplitude as indicated), and paired pulse ratio of amplitude are indistinguishable in Purkinje neurons from WT (n = 13) and P-Rex dKO (n = 12) mice. **B**) Relationship between stimulus strength and EPSC amplitude in cells from WT (n = 10) and P-Rex dKO (n = 10) mice. **C**) Slow EPSCs recorded in response to 10 stimuli at 100 Hz in cells from WT (black trace) and P-Rex dKO (grey trace) mice. Slices were preincubated with 10 µM NBQX to inhibit fast AMPAR currents. Slow current is inhibited by the mGluR antagonist CPCCOEt (20 µM; red trace). Aggregate data shows mean ± s.e.m. amplitude of sEPSC in WT (n = 6) and P-Rex dKO (n = 7) cells, in response to 2, 5 or 10 pulses at 100 Hz. **D**) Current decay after 10 mV depolarizing step (−80 to −70 mV) in representative WT (black trace) and P-Rex dKO (grey trace) cells. Currents are well fitted by biexponential curves (red lines). Aggregate data show no differences in steady-state currents, nor time constants or areas of either exponential phase (n = 6 WT cells; n = 7 P-Rex dKO cells). **E**) Representative current traces recorded from Purkinje neurons during pressure ejection of *s-*AMPA (100 µM) onto the molecular layer (dendrites) or cell somata (soma) of slices from WT and P-Rex dKO mice. Aggregate data show no differences in mean ± s.e.m. amplitudes between genotypes (n = 9 WT and 11 P-Rex dKO cells). **F**) Loose cell-attached recordings of spontaneous action potential firing in representative Purkinje cells from WT and P-Rex dKO mice. Aggregate data show no differences in mean ± s.e.m. for n = 29 cells each.

Using sagittal slices from wild type (WT) and P-Rex1^−/−^/PRex2^−/−^ mice (P-Rex dKO) on a C57B16 genetic background at postnatal days 21–28, we obtained whole cell patch clamp recordings from Purkinje neurons whilst stimulating parallel fibre inputs. Excitatory postsynaptic currents (EPSCs) at parallel fibre synapses showed similar rise times, decay times, and stimulus-response relationships in cells from both strains (**Figure 1A and B**). Parallel fibre synapses also showed similar paired pulse ratios when stimulated with two pulses at 50 ms interval (**Figure 1A**), indicating that short-term plasticity was normal in P-Rex-deficient Purkinje neurons.

We tested for an involvement of P-Rex in the generation of slow mGluR-dependent currents in Purkinje neurons, which requires G-protein coupling to tyrosine phosphatases to open a cation channel [26], [27]. Again, the slow current was indistinguishable between WT and P-Rex dKO mice (**Figure 1C**).

Next, we tested the passive membrane properties of the Purkinje cells by applying a 10 mV hyperpolarizing step, and found that current decay curves from both strains could be well fitted by bi-exponential declines with similar time constants and steady-state (resistive) currents (**Figure 1D**), showing that the cable properties (at the level of a two compartment model) of the P-Rex-deficient cells were normal.

To test for possible gross differences in AMPAR surface expression, we applied *S*-AMPA to slices by pressure ejection in both the molecular layer and at the cell soma. In all cases, currents were smaller at the soma than in the molecular layer, but there were no significant differences between WT and P-Rex dKO cells (**Figure 1E**). Hence, the overall density of AMPAR in dendrites and somata seems unaltered by the P-Rex loss, but with the caveat that the detailed receptor distribution within the membrane, and the relative activation of extrasynaptic and postsynaptic receptors, cannot be deduced through this approach.

Finally, we measured the spontaneous firing rate of Purkinje neurons by loose cell attached recordings, and found that the mean (or median) frequency of spikes, and the distribution of interspike intervals, was indistinguishable between WT and P-Rex-deficient cells (**Figure 1F**).

Hence, by all measures adopted, the excitability and normal synaptic transmission properties of WT and P-Rex-deficient Purkinje neurons were indistinguishable. Despite the defect in dendrite morphology [21], therefore, the essential biophysical properties of Purkinje neurons (as measured at the cell soma) are unaffected by P-Rex-deficiency.

### P-Rex-deficiency causes a defect in a late phase of postsynaptic cerebellar LTP

**Figure 2:**
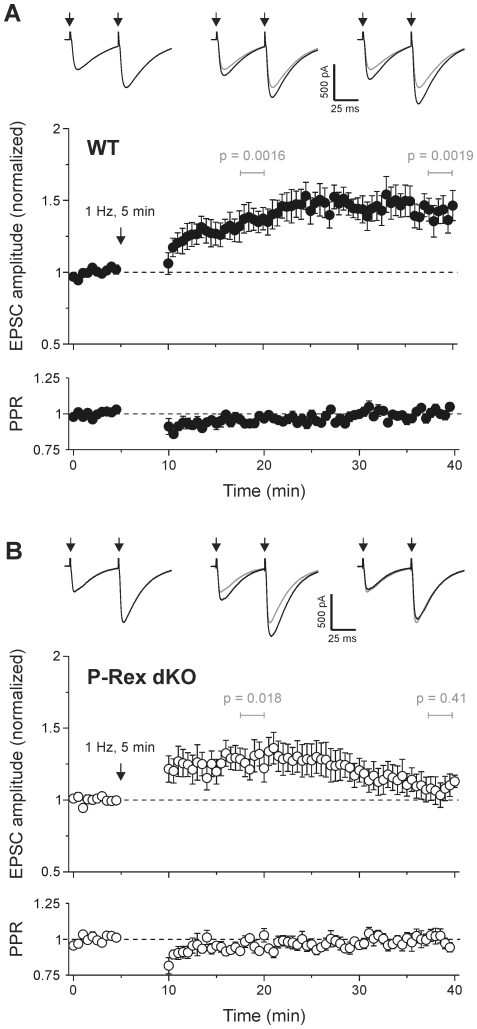
P-Rex is required for a late phase of stimulus-evoked cerebellar LTP. **A**) LTP induced by stimulation of parallel fibres at 1 Hz for 5 min in WT mice. Data are mean normalized amplitude (upper panel) and normalized paired pulse ratio (lower panel) ± s.e.m. for 9 cells. **B**) LTP induced by stimulation of parallel fibres at 1 Hz for 5 min in P-Rex dKO mice is not sustained. Data are mean normalized amplitude (upper panel) and normalized paired pulse ratio (lower panel) ± s.e.m. for 9 cells. Single sample *t* test was used to test for a significant increase in mean amplitude of five consecutive pulses at 10 min and 30 min post 1 Hz tetanus; *p* values were as indicated. Traces show average of 5 consecutive EPSCs evoked by paired pulse stimulation (50 ms interval, at arrows) before (left trace), 10 min after (middle trace) and 30 min after (right trace) 1 Hz stimulation, from a representative cell. Grey traces reproduce EPSC before 1 Hz for comparison.

Next, we tested the effect of P-Rex-deficiency on LTP at the parallel fibre–Purkinje neuron synapse. In cerebellar slices from WT mice, 1 Hz stimulation for 5 min triggered a robust increase in parallel fibre EPSC amplitude that lasted for >30 min (**Figure 2A**), consistent with previous reports [5], [9]. This LTP of synaptic strength did not significantly alter paired pulse ratio (PPR; **Figure 2A**), suggesting that it is a postsynaptic adaptation, as expected [9]. The same stimulus protocol applied to slices from P-Rex dKO mice initially led to an increase in EPSC amplitude similar to that in WT slices (**Figure 2B**), indicating that the induction of cerebellar LTP does not require P-Rex. However, the increase in EPSC amplitude was not sustained: 30 min after the end of the 1 Hz stimulus, the mean amplitude was no longer statistically significantly different from the pre-stimulus amplitude (**Figure 2B**). Hence, P-Rex family enzymes are required for persistent cerebellar LTP. As with WT cells, PPR did not change in P-Rex-deficient cells (**Figure 2B**).

### P-Rex-deficiency causes a defect in a late phase of NO-induced cerebellar LTP

**Figure 3:**
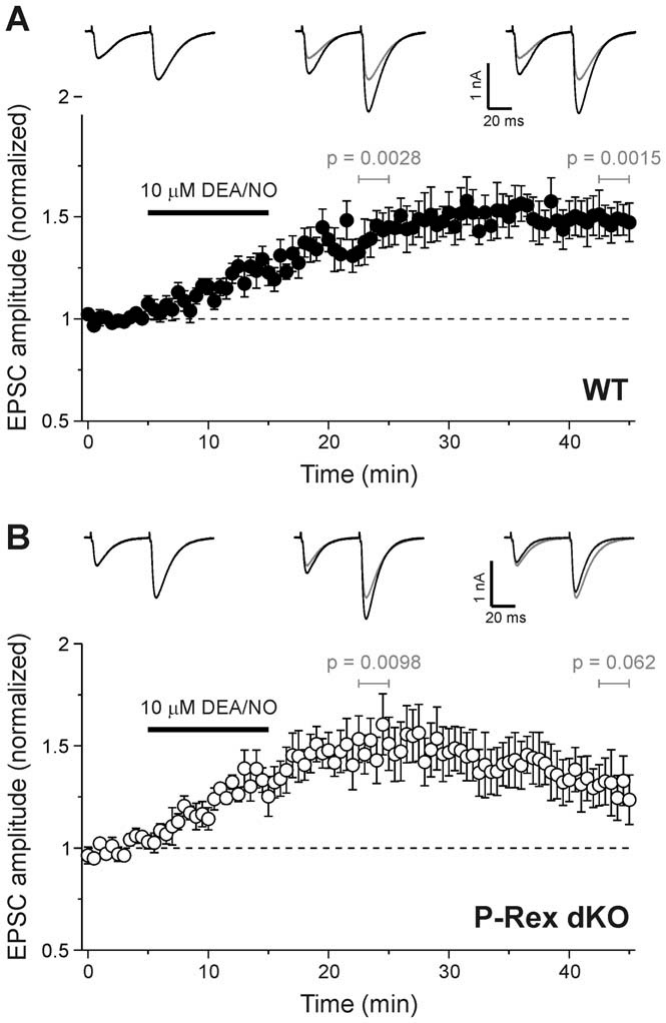
P-Rex is required for a late phase of nitric oxide-induced cerebellar LTP. **A**) Addition of 10 µM DEA/NO to the bath solution for 10 min causes LTP of EPSC amplitude in cells from WT mice. **B**) Potentiation is not sustained in cells from P-Rex dKO mice. Data are mean ± s.e.m. of 6 cells each. Single sample *t* test was used to test for a significant increase in mean amplitude of five consecutive pulses at 10 min and 30 min post DEA/NO washout; *p* values were as indicated. Traces are average of 5 consecutive EPSCs from a representative cell before, 10 min and 30 min after DEA/NO application. Grey traces reproduce EPSC before DEA/NO for comparison.

Parallel-fibre LTP is known to require NO synthesis and can also be evoked by exogenous application of NO donors [9], [11]. Application of the NO donor DEA/NO (10 µM) to WT cerebellar slices led to a robust and sustained increase in parallel fibre EPSC amplitude, similar to that obtained by 1 Hz stimulation (**Figure 3A**). In slices from P-Rex-deficient mice, the DEA/NO-dependent increase in EPSC amplitude was initially similar to that in WT cells, but this potentiation began to decline after removal of NO, such that EPSC amplitude was no longer significantly different (at the p<0.05 level) from pre-stimulus levels after 30 min (**Figure 3B**). Hence, P-Rex family enzymes appear to be required for the maintenance of cerebellar LTP, regardless of whether it is induced by electrical stimulus or by NO donor.

### PI3K inhibition impairs induction of cerebellar LTP

**Figure 4:**
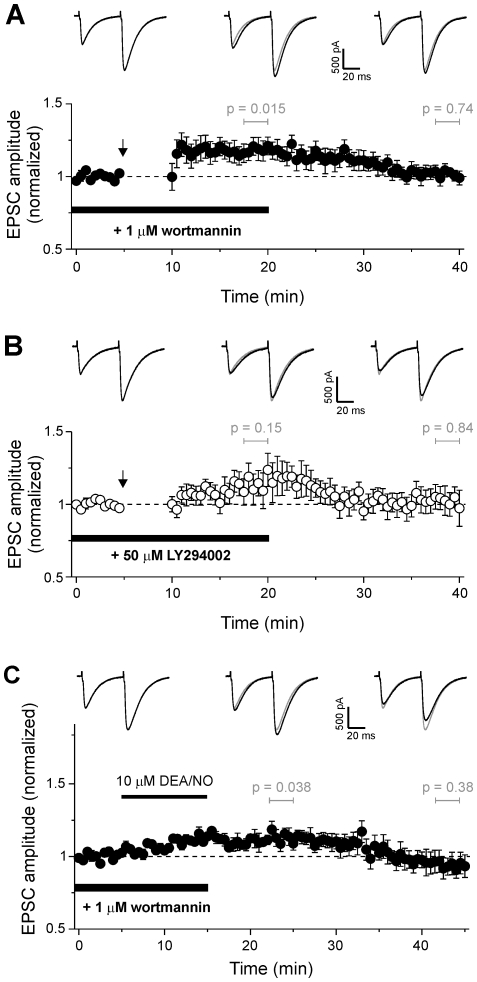
PI3K activity is required for induction of cerebellar LTP. **A**) Application of the irreversible PI3K inhibitor wortmannin (1 µM; n = 9 cells) to the bath from 10 min before until 10 min after 1 Hz stimulation for 5 min (arrow) reduces the amplitude and duration of LTP. **B**) Application of the reversible PI3K inhibitor LY294002 (50 µM; n = 8) to the bath from 10 min before until 10 min after 1 Hz stimulation for 5 min (arrow) blocks LTP induction. Data are mean ± s.e.m. Single sample *t* test was used to test for a significant increase in mean amplitude of five consecutive pulses at 10 min and 30 min post LTP-evoking tetanus; *p* values were as indicated. Traces are averages of 5 consecutive EPSCs from a representative cell before, 10 min and 30 min after 1 Hz stimulation. Grey traces reproduce EPSC before 1 Hz for comparison. **C**) Addition of wortmannin to the bath solution for 10 min before and throughout application of 10 µM DEA/NO blocks LTP. Data are mean ± s.e.m. of 6 cells. ns = mean amplitude is not significantly different from 1 (p>0.05, single sample *t* test). Traces are average of 5 consecutive EPSCs from a representative cell before, 10 min and 30 min after DEA/NO application. Grey traces reproduce EPSC before DEA/NO for comparison.

P-Rex is activated by PtdIns(3,4,5)P_3_, the product of class 1 PI3K activity [28]. We therefore tested whether PI3K also plays a role in cerebellar LTP. A vehicle control (0.1% DMSO) showed the same pattern of LTP as untreated WT cells, with EPSC amplitude increasing progressively after 1 Hz stimulation and remaining potentiated for >30 min (p = 0.0076 at 27.5–30 min, n = 9 cells; data not shown). In contrast, in slices preincubated with the irreversible PI3K inhibitor wortmannin [29] (1 µM applied 10 min before until 10 min after period of 1 Hz stimulation), the increase in EPSC amplitude was reduced (initial mean amplitude 1.21±0.06 s.e.m. at 10 min post-stimulus, normalized to pre-stimulus amplitude; cf. 1.38±0.07 s.e.m. for control), and thereafter declined to pre-stimulus levels (**Figure 4A**), suggesting that PI3K signalling contributes to the induction of cerebellar LTP.

We repeated these experiments with an alternative, reversible PI3K inhibitor that is more stable in aqueous solution than wortmannin, LY294002 (50 µM) [29]. LY294002 treatment blocked LTP entirely. EPSC amplitude did not significantly increase at any time after 1 Hz stimulation (**Figure 4B**). Hence, PI3K activity is required for induction of cerebellar LTP.

The same dependence of cerebellar LTP on PI3K activity was also observed when LTP was induced with DEA/NO, as preincubation of slices with 1 µM wortmannin for 10 min impaired induction of potentiation by DEA/NO (**Figure 4C**).

**Figure 5:**
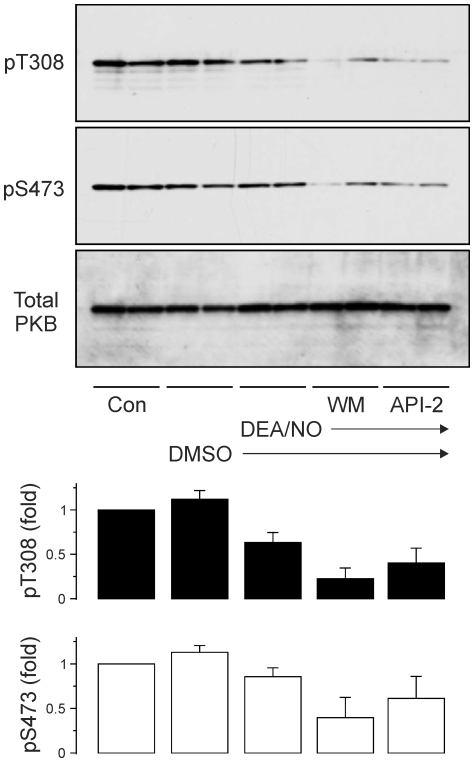
PKB phosphorylation in cerebellar slices. Cerebellar slices from WT mice were treated with 1 µM wortmannin or vehicle control (DMSO) for 10 min or with API-2 for 30 min in their recovery chamber before addition of 10 µM DEA/NO for another 10 min. Total lysates were prepared and subjected to Western blotting with phospho-S473 and phospho-T308 specific PKB antibodies as well as total PKB antibodies as described in Materials and Methods. Blots shown are from one experiment representative of three. Bar graphs show densitometric analysis of PKB phosphorylation on T308 or S473, as indicated, compared to levels in untreated “control” samples and are mean ± s.e.m. of three experiments.

To test whether DEA/NO treatment might be a sufficient signal to stimulate PI3K activity in cerebellar slices, we measured PI3K activity indirectly by Western blotting for phosphorylation of the PI3K target protein kinase B (PKB/Akt) in total lysates of cerebellar slices. We found substantial levels of basal PKB phosphorylation, both on T308 and S473, which suggested high tonic PI3K activity in the slices. 10 µM DEA/NO did not increase the basal PKB activity any further (**Figure 5**). Pre-incubation with the PI3K inhibitor wortmannin (1 µM), or the PKB inhibitor API-2 (1 µM), substantially reduced PKB phosphorylation levels (**Figure 5**). These results suggest that tonic activation of PI3K under resting conditions is substantial, and that treatment with DEA/NO did not detectably increase global PKB activation in the slice.

### Rac and PKB activity are required for a late phase of cerebellar LTP

**Figure 6:**
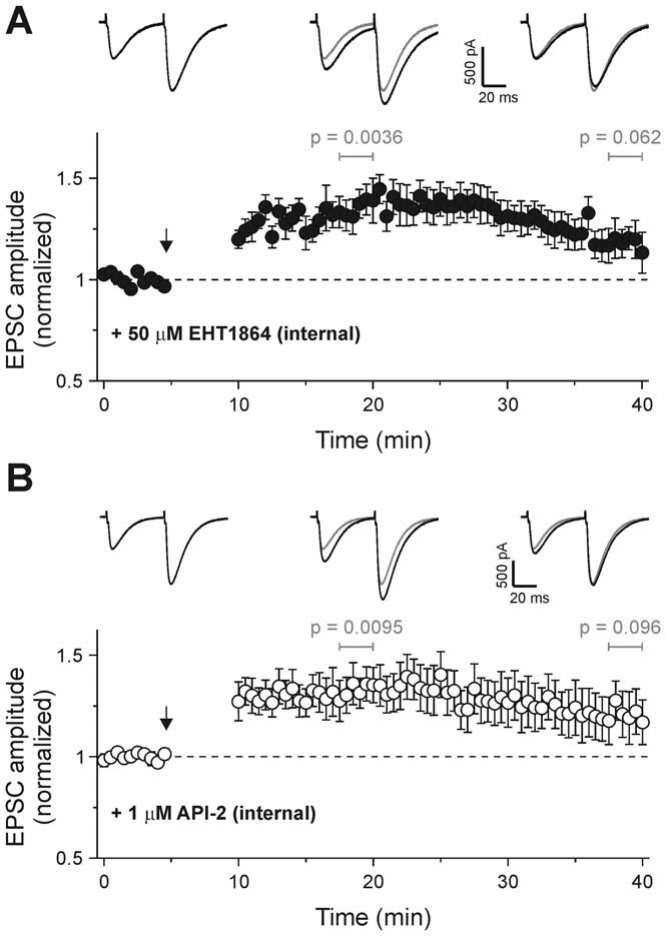
Rac and PKB activities are required for a late phase of cerebellar LTP. **A**) Addition of the Rac inhibitor EHT1864 (50 µM; n = 7) to pipette solution decreases LTP duration. **B**) The PKB inhibitor API-2 (1 µM; n = 8) added to the pipette solution also decreases LTP duration. Arrows indicate start of 1 Hz stimulation for 5 min. Data are mean ± s.e.m. Single sample *t* test was used to test for a significant increase in mean amplitude of five consecutive pulses at 10 min and 30 min post 1 Hz tetanus; *p* values were as indicated. Traces are averages of 5 consecutive EPSCs from a representative cell before, 10 min and 30 min after 1 Hz stimulation. Grey traces reproduce EPSC before 1 Hz for comparison.

The downstream target of P-Rex, Rac, has been implicated in hippocampal LTP [18]. We therefore tested for a role of Rac in cerebellar LTP with the recently developed compound EHT1864, which has been used to inhibit Rac-dependent processes in several cell types [30]. We found a similar pattern of potentiation to that in cells from P-Rex-deficient mice. EPSC amplitude was initially increased by 1 Hz stimulation, but the potentiation was not sustained over 30 min (**Figure 6A**). Hence, it is plausible that Rac activity could control the maintenance of cerebellar LTP downstream of P-Rex.

As inhibition of PI3K impaired the induction of cerebellar LTP, it seemed likely that other PtdIns(3,4,5)P_3_ downstream targets control LTP in addition to P-Rex. PKB is the archetypical PI3K target (although more than 50 are known altogether), and in hippocampal pyramidal neurons, PKB activation downstream of PI3K has been implicated in the blockade of LTD during LTP [31]. We therefore tested the effect of the PKB inhibitor API-2 on LTP [32]. We found the same effect as P-Rex deletion or Rac inhibition: API-2 allowed the induction of LTP but prevented the sustained potentiation of EPSC amplitude (**Figure 6B**). Hence, while PKB is not required for LTP induction, it controls, like P-Rex, a later phase of cerebellar LTP.

## Discussion

Postsynaptic LTP at the parallel fibre-Purkinje cell synapse has only recently been described [5], [9], and the molecular basis of this form of plasticity is poorly understood – certainly in comparison to the converse phenomenon, LTD [8]. The induction mechanism of LTP involves modest elevation of postsynaptic Ca^2+^ to activate protein phosphatases [10], in combination with NO acting in a cGMP-independent manner [11]. This mechanism distinguishes cerebellar LTP from classical LTP in the hippocampus (reviewed in [12]).

Hippocampal LTP occurs in several overlapping phases, and the signalling pathways involved in early and later phases differ. We sought to explore the possibility that cerebellar LTP also exhibits such early and late phases, and have identified a signalling pathway linking PI3 kinase activity to Rac activation via the guanine exchange factor P-Rex, which is necessary for the sustained expression of cerebellar LTP for >30 min.

### P-Rex

P-Rex is a guanine nucleotide exchange factor (GEF) for the small GTPase Rac. Two P-Rex isoforms exist, P-Rex1 and P-Rex2, as well as a splice variant (P-Rex2b) that is expressed only in the heart. P-Rex family enzymes are best known for their roles in leukocytes, although an important role in cancer is emerging [28], [33], [34].

In the brain, P-Rex1 is widely expressed, whereas P-Rex2 is expressed selectively in Purkinje cells [21]. The primary dendrites of P-Rex2-deficient Purkinje neurons are slightly narrower, shorter and more convoluted than those of wild type cells. P-Rex2-deficient mice also exhibit mild motor coordination defects which worsen with age, particularly in females. Deficiency in both P-Rex1 and P-Rex2 does not disrupt Purkinje cell dendrite morphology further, but causes more severe motor defects, with gait and posture abnormalities as well as motor coordination impairment [21]. Surprisingly, we found that the defect in dendrite morphology did not result in a detectable change in the passive membrane properties of Purkinje neurons, the somatic or dendritic AMPAR densities, the kinetics of synaptic transmission, or the short-term plasticity of parallel fibre–Purkinje cell synapses. This suggests either that the anatomical differences in P-Rex-deficient cells are too minor to influence the cable properties of the cells, or that the expression and distribution of ion channels has been adjusted by compensatory mechanisms such that the normal input-output properties of the dendritic tree are maintained.

In contrast to the lack of effect on excitability and normal synaptic transmission, we found that deletion of P-Rex impairs a late phase of LTP at parallel fibre to Purkinje neuron synapses. Potentiation of EPSC amplitude evoked by 1 Hz stimulation or by exogenous NO is not sustained in P-Rex-deficient mice. The implication is that an initial induction process that shifts the protein kinase/phosphatase equilibrium to favour increased AMPA receptor density must be reinforced by signalling pathways connected to P-Rex. This may either reflect a failure to maintain transient LTP, or a failure to induce a second, late phase of LTP that takes over from the earlier phase of potentiation.

### Rac

The known role of P-Rex is as a Rac-GEF, and application of a drug (EHT1864) known to inhibit Rac-dependent processes had a similar impact on LTP duration as P-Rex elimination. Rac regulates many cellular processes, including vesicle trafficking, gene transcription and generation of reactive oxygen species, any of which may in principle play a role in plasticity mechanisms [35]. However, Rac is also an essential controller of cytoskeletal re-arrangements in many cells [36], and its perturbation leads to large-scale anatomical defects during Purkinje neuron maturation [37]. In the hippocampus, late phase LTP is associated with activation of several Rho GTPases, including Rac, leading to actin polymerization and the stabilization of changes in spine morphology [20].

In contrast to our results, inhibition of Rac during and after LTP induction does not cause a failure of LTP maintenance in the hippocampus [20]. Instead, Rac inhibition increases the vulnerability of LTP to reversal by an actin polymerization inhibitor, latrunculin, suggesting that it acts to stabilize actin filaments after assembly. Our results with a Rac inhibitor were analogous to results with a RhoA inhibitor on hippocampal LTP [20], suggesting that the small GTPases involved in the (multi-step) cytoskeletal re-arrangement associated with late phase LTP may differ between pyramidal and Purkinje neurons.

The scope of structural remodelling associated with LTP is unclear. In the hippocampus, both local changes in spine ultrastructure, and large-scale changes in spine morphology have been described in response to LTP induction [38]. In the cerebellum, complex motor tasks have been shown to increase spine density in Purkinje neurons [39], suggesting a possible physical correlate for synaptic plasticity. However, LTD is not associated with a diminution of spine volume or number [40].

### PI3 kinase

P-Rex is substantially activated by the lipid second messenger PtdIns(3,4,5)P_3_, which is generated by the activity of phosphoinositide 3-kinase (PI3K), suggesting that PtdIns(3,4,5)P_3_ may be an upstream trigger for enhancing Rac activity. However, inhibition of PI3K abolished LTP entirely, implicating PtdIns(3,4,5)P_3_ in the initial induction of LTP. LY294002 is a reversible PI3K inhibitor, but suppression of LTP lasted after washout of the drug, suggesting that PI3K inhibition does not simply mask expression of LTP, as observed in the hippocampus [23]. These results therefore imply that PI3K may play multiple roles in LTP, and that P-Rex is only one of the downstream targets engaged by PtdIns(3,4,5)P_3_. PKB appears to be a second target, as inhibition with API-2 had a similar effect on late phase LTP as P-Rex deletion, but the failure of PKB and P-Rex inhibition to block induction suggests that additional PtdIns(3,4,5)P_3_ targets have a role in LTP induction.

PtdIns(3,4,5)P_3_ synthesis is catalysed by class 1 PI3Ks, which fall into two types: 1A which are activated by protein tyrosine kinases, and 1B which are activated by Gβγ subunits. Both types are cooperatively activated by binding of activated Ras [41]. Candidate receptors for activation of class 1A or class 1B PI3Ks abound: receptor tyrosine kinases for ephrins and neurotrophins, and GPCRs for glutamate, GABA, purines and monoamines, are known to be expressed by Purkinje neurons, and can regulate synaptic transmission [8]. At this stage, it is unclear whether PI3Ks are switched on by plasticity-inducing stimuli, or if tonic activation of upstream receptors provides a permissive signal for induction and maintenance of LTP by other signalling pathways. The high basal levels of PKB phosphorylation in cerebellar slices (**Figure 5**) suggest that tonic activation is plausible.

In many cell types and responses, PtdIns(3,4,5)P_3_ synthesis acts as a spatial signal, drawing proteins that contain a PH domain to regions of the membrane where PI3K activity is high [41]. We are not aware of any evidence in the literature that P-Rex or PKB enhance protein phosphatase activity, which would be a simple mechanism by which their role in LTP could be understood. Similarly, a pathway linking NO synthesis with PI3K could also fit with existing knowledge of the mechanisms of LTP induction, especially as PKB is known to promote NO synthesis by eNOS [42], [43]. However, LTP evoked by exogenous application of NO was also vulnerable to PI3K inhibition, indicating that putative PtdIns(3,4,5)P_3_-dependent targets must operate either downstream or independently of NO synthesis.

### Summary

We have reported the first evidence for different phases of LTP in the cerebellum, where the late phase depends on the activation of a PI3K-P-Rex-Rac signalling pathway. The observations that the alternative PI3K target PKB also plays a role in late phase LTP, and that PI3K inhibition blocks LTP induction, suggest that more PI3K-dependent control points in the mechanism of cerebellar plasticity remain to be determined. It seems probable that, as with hippocampal LTP, multiple as-yet unidentified signalling pathways will play a role in the induction, expression and maintenance of cerebellar LTP.

## Materials and Methods

### Ethics statement

Study protocols complied with UK Home Office (project licence numbers 80/1875 and 80/2335) and local ethics committee (Babraham Research Campus Animal Welfare, Experimentation, and Ethics Committee approval reference numbers 0704 and 1009) guidelines, and were carried out under UK Home Office Certificate of Designation number PCD 80/4804.

### Materials

(*s*)-AMPA, API-2, bicuculline methiodide, CPCCOEt, NBQX and picrotoxin were from Tocris (Bristol, UK). EHT1864, LY294002, and wortmannin were from Sigma. DEA/NO was from Axxora (Nottingham, UK).

### Mice

P-Rex1^−/−^/P-Rex2^−/−^ have been described before and were backcrossed a minimum of 7 times to C57B16 genetic background [21]. They were bred and housed in a shower-in barrier facility in open caging. They were crossed with C57B16 mice at least once every two years to minimise genetic drift. C57Bl6 mice were used as WT controls. Male and female mice between 21 and 28 days of age were used for experiments.

### Preparation of cerebellar slices

Sagittal slices (200 µm) were prepared from the cerebellar vermis. Animals were rendered unconscious with CO_2_ and killed by cervical dislocation. After decapitation, the cerebellum was removed rapidly and placed into ice cold (4–7°C) buffer containing NaCl (126 mM), KCl (3 mM), NaH_2_PO_4_ (1.2 mM), NaHCO_3_ (25 mM), glucose (15 mM), MgSO_4_ (3 mM) and CaCl_2_ (0.5 mM), gassed with carbogen (95% O2/5% CO_2_). Cerebella were cleaned of meninges and cut with a vibratome (VT1000S, Leica, Milton Keynes, UK), before being transferred to a recovery chamber containing: NaCl (126 mM), KCl (3 mM), NaH_2_PO_4_ (1.2 mM), NaHCO_3_ (25 mM), glucose (15 mM), MgSO_4_ (2 mM) and CaCl_2_ (2 mM), and continuously gassed at 32°C for 1 h. The chamber was then allowed to cool to room temperature for at least 30 min, before slices were used for experiments over the next 4–6 hours.

For recording, slices were transferred to an immersion chamber and perfused with a bath solution containing NaCl (126 mM), KCl (3 mM), NaH_2_PO_4_ (1.2 mM), NaHCO_3_ (25 mM), glucose (15 mM), MgSO_4_ (2 mM) and CaCl_2_ (2 mM), gassed with carbogen and supplemented with either 20 µM bicuculline methiodide or 20 µM picrotoxin to inhibit GABA_A_ receptors.

### Electrophysiology

Recording electrodes were manufactured from borosilicate glass, and filled with an internal solution containing (mM): K-gluconate (110), KCl (5), HEPES (50), EGTA (0.05), MgSO_4_ (4), ATP (4), GTP (0.2), phosphocreatine (9), pH to 7.4 with 1 M KOH. Whole cell voltage clamp recordings (holding potential = −70 mV) were made from visually identified Purkinje neuron somata in the Purkinje cell layer using an Axopatch 200B amplifier (Molecular Devices, Wokingham, UK) as previously described [44]. Currents were low pass filtered at 4–5 kHz, and sampled at 25 kHz, using a micro1401 A/D convertor and Spike 2 software (CED, Cambridge, UK). Series resistances ranged from 5–15 MΩ, and were compensated by >85%. Stimulating electrodes were also pulled from borosilicate glass, and filled with bath solution with series resistance of 1–2 MΩ, connected to a constant current isolated stimulator (Digitimer, Welwyn, UK). Parallel fibres were stimulated with pairs of pulses (50 ms interval) at 0.033 Hz, using a patch electrode filled with bath solution and positioned in the molecular layer, connected to an isolated constant current stimulator (5–50 µA, 80 µs). To induce LTP, stimulation frequency was raised to 1 Hz for 5 min, and cells were switched to current clamp (I_h_ = 0).

### Western blotting

Cerebellar slices, prepared as described above, were treated with 1 µM wortmannin or vehicle control for 10 min or with API-2 for 30 min in their recovery chamber before addition of DEA/NO for another 10 min. The slices were then transferred rapidly into 1 ml boiling 1.2× Laemmli SDS-PAGE sample buffer, homogenised by trituration with a syringe and 25-gauge needle, and further boiling. Total lysates were subjected to Western blotting with phospho-S473 or phospho-T308 specific PKB antibodies (Cell Signalling Technologies). Membranes were then glycine-stripped (25 mM glycine, pH 2.0, 1% SDS, 30 min at 22°C) and re-blotted with total PKB antibodies (Cell Signalling Technologies). Blots were scanned and subjected to densitometric analysis using NIH ImageJ.

### Analysis

EPSC traces shown are the average of 5 sequential recordings. Aggregate data are the mean ± s.e.m. of multiple cells as indicated in figure legends. Statistical significance of normalized data was tested for by single sample Student's *t* test. Mean normalized amplitude for each cell was calculated for five consecutive pulses at 7.5–10 min and 27.5–30 min after the end of the LTP-inducing stimulus. Any difference from 1 in the mean amplitude (for the population of cells) was considered significant if p<0.05. Cells were excluded from the analysis if series resistance changed by more than 5 MΩ, or if the standard deviation of the pulse-to-pulse variation in amplitude was >0.1 during the baseline period.
